# Immune Aging and Trauma Outcomes: Admission Inflammatory Profiles Consistent with Immunosenescence Are Associated with Excess One-Year Mortality Despite Similar in-Hospital Mortality in Older Adults with Multisystem Trauma

**DOI:** 10.3390/biology15141151

**Published:** 2026-07-15

**Authors:** Furkan Türkoğlu, Ahmet Gedik, Berk Koncalıoğlu, Elif Nur Gencer, Necip Gökhan Güner, İlteriş Türk, Batuhan Gencer, Özgür Doğan

**Affiliations:** 1 Department of General Surgery, Aktif International Hospital, 41275 Kocaeli, Türkiye; drfurkanturkoglu@gmail.com; 2 Department of Medical Pharmacology, Ataturk University, 25050 Erzurum, Türkiye; ahmetgedik@me.com; 3 Department of Orthopaedics and Traumatology, Sultanbeyli State Hospital, 34935 İstanbul, Türkiye; berkkoncalioglu@gmail.com; 4 Department of General Surgery, Tuzla State Hospital, 34947 İstanbul, Türkiye; elifnurkocmar@yahoo.com; 5 Department of Emergency Medicine, Sakarya University Training and Research Hospital, 54100 Sakarya, Türkiye; necipguner@sakarya.edu.tr; 6 Department of Thoracic Surgery, Ankara Etlik City Hospital, 06170 Ankara, Türkiye; turkilteris@gmail.com; 7 Department of Orthopaedics and Traumatology, Marmara University Pendik Training and Research Hospital, 34722 İstanbul, Türkiye; 8 Department of Orthopaedics and Traumatology, Ankara Bilkent City Hospital, 06800 Ankara, Türkiye; dr.ozgurdogan@gmail.com

**Keywords:** multisystem trauma, geriatric trauma, immunosenescence, one-year mortality, in-hospital mortality, neutrophil-to-lymphocyte ratio, injury severity score, lymphocyte count, creatinine, hypoalbuminemia

## Abstract

Objectives: Aging is associated with immunosenescence, which may alter the physiological response to trauma and influence long-term outcomes. This study aimed to compare clinical and laboratory parameters associated with in-hospital (iHM) and one-year mortality (OYM) between adult and geriatric patients with multisystem trauma and to explore laboratory findings consistent with immunosenescence in geriatric patients. Methods: In this retrospective study, 345 patients with multisystem trauma were categorized into adult (*n* = 255) and geriatric (*n* = 90) groups according to age. Clinical characteristics and laboratory parameters obtained at emergency department admission were analyzed in relation to iHM and OYM. Results: Although iHM did not differ significantly between groups (*p* = 0.764), OYM was significantly higher in the geriatric group (*p* < 0.001). In adults, iHM was associated with a higher Injury Severity Score (ISS), prolonged intensive care unit (ICU) stay, elevated white blood cell and monocyte counts, and hypoalbuminemia. OYM in adults was further associated with a higher ISS, longer hospital stays, and lower hemoglobin and hematocrit levels. In geriatric patients, iHM was associated with a higher ISS, male sex, high-energy trauma, elevated lymphocyte and creatinine levels, and lower hematological ratios. OYM in the geriatric group was additionally associated with advanced age, major surgical intervention, thrombocytopenia, elevated urea–creatinine levels, hypoalbuminemia, and lower hematological ratios. Conclusions: Despite comparable in-hospital mortality, geriatric patients with multisystem trauma demonstrated significantly higher one-year mortality. The reduced post-traumatic inflammatory response observed in geriatric patients may be indicative of underlying immunosenescence and diminished immune reserve, thus emphasizing the necessity for age-specific prognostic models in trauma care.

## Simple Summary

As people age, their immune system undergoes gradual changes, a process known as immunosenescence. This means that the body’s defense system may respond differently to severe injury than it does in younger adults. In this study, we compared younger and older adults with serious injuries affecting more than one part of the body and analyzed routine blood test results obtained when they first arrived at the emergency department. Although death during the initial hospital stay was similar in both age groups, older adults were much more likely to die within one year after injury. We also found that several routine blood test results were associated with survival in different ways in older and younger patients. While stronger inflammatory responses are often linked to poor outcomes in younger adults, older patients who died frequently showed lower inflammatory blood test values, suggesting a different biological response to severe injury. These findings do not prove that immunosenescence is responsible for these differences, but they are consistent with age-related changes in immune function. A better understanding of these changes may support future research and ultimately improve the long-term care and follow-up of older patients after severe trauma.

## 1. Introduction

Trauma remains one of the leading causes of death, disability, and healthcare-related burden worldwide. While major advances in prehospital care, damage-control strategies, and intensive care management have improved early survival rates among severely injured patients, the epidemiology of trauma is changing. Population aging has resulted in a substantial increase in the number of elderly patients presenting with major trauma, creating an increasing challenge for trauma systems globally. In this age group, outcomes are often poorer, with mortality rates exceeding those of younger patients, even when the severity of the injury appears comparable. This disparity highlights the distinct biological vulnerability of elderly patients and the importance of specialized management approaches [1]. Furthermore, predicting secondary complications is challenging in geriatric patients because aging modifies baseline physiology, blunts inflammatory responses, and may mask early clinical deterioration [2].

Geriatric patients are a particularly vulnerable group with injury responses that differ substantially from those of younger adults. While the Injury Severity Score (ISS), first described by Baker et al. [3], remains fundamental for quantifying anatomical trauma, a similar degree of structural injury can lead to significantly different physiological outcomes in older individuals. This discrepancy is largely due to diminished physiological reserves and the high prevalence of chronic comorbid conditions, which together contribute to frailty syndrome. In addition to serving as a pre-existing vulnerability, frailty has a decisive influence on recovery after trauma and is increasingly recognized as a major driver of prolonged morbidity and late mortality in the geriatric population [4].

A key factor in predicting trauma-related outcomes is the systemic immune response. In older people, immune systems are shaped by age-related changes collectively known as immunosenescence. These changes are often accompanied by inflammaging, a persistent state of low-grade inflammation. Together, these processes create a dysregulated immune environment that weakens host defense mechanisms and disrupts metabolic homeostasis [5]. Emerging evidence suggests that, in elderly patients, baseline immune aging may influence the reactivity of circulating leukocytes more than trauma or surgery itself, thereby attenuating the acute-phase response normally expected after injury [6].

Accordingly, applying conventional adult biomarker thresholds directly to geriatric patients may be misleading. Commonly used hematological indices, such as the neutrophil-to-lymphocyte (NLR), monocyte-to-lymphocyte (MLR), and platelet-to-lymphocyte (PLR) ratios, are substantially affected by age-related immune and physiological alterations. Previous research has extensively examined the prognostic significance of these hematological markers and ratios in both adult and elderly patients [7,8,9]. In elderly trauma patients, elevated or dysregulated NLR patterns at admission appear to reflect heightened physiological stress together with limited compensatory capacity and have been associated with an increased risk of adverse early outcomes, including unexpected mortality such as sudden cardiac arrest [10]. Furthermore, preoperative NLR has been identified as an independent prognostic indicator of long-term mortality in very elderly trauma patients with severe fractures, suggesting its potential value in risk stratification in this population [11]. Similarly, polytrauma has been the subject of extensive research, with a significant body of research focusing on prognostic indicators and predictors of mortality [12,13,14,15,16]. However, comparatively fewer studies have specifically evaluated the prognostic significance of admission biological parameters in multisystem trauma populations. Furthermore, the extent to which these parameters can be directly compared in terms of their prognostic value in adult and geriatric multisystem trauma patients remains limited. In view of the age-related immune and physiological alterations associated with immunosenescence, further evidence is required to determine whether the prognostic implications of commonly used laboratory biomarkers differ between adult and older trauma patients.

Accordingly, this retrospective, multidisciplinary study was designed to compare clinical and laboratory predictors of in-hospital mortality (iHM) and one-year post-injury mortality (OYM) in adult and geriatric trauma patients, and to explore laboratory findings consistent with immunosenescence in geriatric patients. By analyzing physiological variables, biochemical parameters, and complete blood count indices obtained at initial emergency department admission, this study aims to clarify age-dependent differences regarding mortality in the immune-metabolic response to trauma.

## 2. Materials and Methods

### 2.1. Study Design and Ethical Approval

This retrospective, single-center observational study included a cohort of trauma patients who were evaluated and treated in the emergency department of the study hospital during the specified dates. The study protocol was approved by the Ankara City Hospital Ethics Committee for Clinical Research No. 2 on 25 March 2021, under the code E2-21-266. This observational study was conducted in accordance with the ethical standards outlined in the Declaration of Helsinki [17], and reporting was performed in accordance with the Strengthening the Reporting of Observational Studies in Epidemiology (STROBE) statement and guidelines [18].

### 2.2. Patient Selection

From 2018 to 2021, all patients aged 18 years and older who were admitted to the study hospital, which is a tertiary trauma center, and treated for multiple traumatic injuries were screened for inclusion in the study. For the purposes of this study, the term “patient with multisystem trauma” was operationally defined as the presence of traumatic injuries affecting at least three distinct anatomical regions. Consequently, patients with an Abbreviated Injury Scale (AIS) score of at least 1 in a minimum of at least three distinct anatomical systems were deemed eligible for inclusion. Patients with an initial ISS of 75, those declared deceased on arrival, and those brought to the emergency department in cardiopulmonary arrest who did not respond to cardiopulmonary resuscitation were excluded from the study. Furthermore, patients with incomplete or unavailable demographic, clinical, or laboratory data were excluded from the study. Furthermore, patients for whom reliable one-year mortality data were not available were excluded from the final analysis. Following the implementation of predetermined inclusion and exclusion criteria, a total of 345 patients were deemed eligible and were included in the study cohort of this retrospective analysis. After the selection of the patient cohort and the determination of its characteristics, an investigation was conducted into the presence of age-related differences in mortality-associated factors. To this end, patients were stratified into two predefined age groups according to their age at the time of admission. The adult population is delineated as individuals between the ages of 18 and 64. The geriatric group is defined as individuals who are at least 65 years of age. The age threshold of 65 years was chosen because it is the most widely accepted definition of the geriatric population in trauma research. It is also commonly used in major trauma registries and clinical guidelines, which facilitate comparison with previous studies [19,20].

### 2.3. Outcome Measurements

The investigation encompassed both adult and geriatric cohorts, with a focus on the evaluation of in-hospital mortality and one-year mortality after the initial trauma. In-hospital mortality was defined as death occurring during continuous hospitalization following the initial trauma admission to the index hospital and before discharge, regardless of the total duration of hospitalization. The in-hospital mortality data were collected retrospectively from two sources: the institutional hospital information system and the independent archival records of the relevant clinical departments.

The other primary endpoint of this study was one-year mortality following initial trauma. One-year mortality was defined as all-cause mortality occurring within one calendar year from the date of the initial trauma admission to the index hospital, irrespective of the underlying cause of death. One-year mortality data were obtained from the national healthcare database maintained by the Turkish Ministry of Health. Mortality status was verified through integrated records from the hospital information system and the centralized electronic health record platform, allowing complete ascertainment of one-year survival status for all eligible patients following the index trauma. Accordingly, no patients were excluded because of missing one-year mortality data.

To evaluate the potential influence of patient inclusion on the observed associations, a sensitivity analysis was performed. This analysis involved restricting the cohort to patients with an Injury Severity Score (ISS) > 15, a threshold that is commonly used to define severe trauma. In this particular subgroup, a re-evaluation of all laboratory parameters and CBC-derived ratios was conducted, with a focus on their association with both in-hospital and one-year mortality. The objective of this analysis was to evaluate the robustness and consistency of the primary findings, as opposed to providing a secondary descriptive comparison. Consequently, the focus is limited to the presentation of statistical significance (*p* values) from the subgroup analyses.

### 2.4. Demographic and Clinical Parameters

A comprehensive set of demographic characteristics was systematically documented for all patients, encompassing age, sex, injury energy, Injury Severity Score, and length of stay (LOS) in hospital and in intensive care unit (ICU) during the initial admission to the index hospital. Furthermore, the necessity for major surgery at the initial presentation was meticulously recorded. Most major surgical procedures were open reductions and internal fixation of fractures in the upper and lower extremities. Less frequently performed procedures included exploratory laparotomy for acute abdominal injuries, neurosurgical interventions for traumatic intracranial hemorrhages, and urological procedures for renal trauma.

The clinical and demographic data of the entire cohort of patients were obtained retrospectively from two sources: the institutional hospital record system and the independent archival records of the relevant clinical departments. To ensure methodological consistency, the analysis was limited to findings documented during the initial emergency department presentation and index hospitalization. Consequently, the study did not consider parameters related to subsequent hospital admissions, readmissions after discharge, or data obtained during later inpatient follow-up.

The categorization of injury energy was determined by the mechanism documented at the time of admission. Low-energy injury was defined as injuries resulting from mechanisms such as simple falls or other comparable low-impact events. The term “high-energy injury” was defined as injuries resulting from mechanisms such as motor vehicle collisions, falls from height, gunshot wounds, or other major blunt trauma mechanisms. In evaluating the necessity for major surgical intervention during the initial admission, only operative procedures performed in the operating room under general, spinal, or local anesthesia were classified as major surgery. All procedures conducted in the emergency department were classified as minor surgical procedures and were not recorded as major surgical procedures. The range of procedures encompassed, but was not limited to, the following: reduction in fractures or dislocations; irrigation and primary closure of open wounds; tube thoracostomy; urinary catheterization; and similar bedside interventions.

### 2.5. Laboratory Parameters

All laboratory parameters included in the study were obtained from the first blood samples collected during the initial evaluation in the emergency department immediately after admission. If the initial sample was unsuitable for analysis because of clotting or insufficient volume, the first repeat sample collected during the same emergency department evaluation was used instead.

The following laboratory parameters were recorded for all patients at the time of initial admission: hemoglobin (HGB), hematocrit (HCT), platelet count (PLT), leukocyte count (WBC), neutrophil count (Neu), lymphocyte count (LYM), monocyte count (Mono), serum urea, serum creatinine (Cr), and serum albumin (Alb). Subsequently, inflammation-based hematological indices were derived from complete blood count parameters. These indices included the neutrophil-to-lymphocyte ratio (NLR), monocyte-to-lymphocyte ratio (MLR), and platelet-to-lymphocyte ratio (PLR). To maintain methodological consistency, serial laboratory measurements obtained during hospitalization, as well as laboratory parameters from subsequent follow-up assessments, were not included in the analysis.

### 2.6. Statistical Analysis

Statistical analyses were performed using IBM SPSS Statistics for Windows, version 26.0 (IBM Corp., Armonk, NY, USA). The distribution characteristics of continuous variables were assessed using both the Kolmogorov–Smirnov test and visual inspection methods, including evaluations of histograms and probability plots. Given the presence of non-normal distributions among all continuous variables, a median (minimum–maximum) approach was employed for data summarization. Categorical variables were presented as numbers (n) and percentages (%). The statistical analysis of categorical variables was performed using the Chi-square test or Fisher’s exact test, as appropriate. To compare continuous variables between two independent groups, the Mann–Whitney U test was employed. Subgroup analyses related to mortality were conducted separately for the adult and geriatric cohorts for both in-hospital mortality and one-year post-traumatic mortality. A two-tailed *p* value less than 0.05 was considered statistically significant.

## 3. Results

The median age of the 345 multisystem trauma patients included in the analysis was 49 years (range: 18–95 years). The sex distribution was 116 females (33.6%) and 229 males (66.4%), while the age group distribution was 255 adults (73.9%) and 90 geriatric (26.1%) patients. The median ISS was 13 (range: 4–57). The in-hospital mortality rate during the initial admission was 4.1% (*n* = 14 patients), whereas the one-year post-injury mortality rate was 7.8% (*n* = 27 patients). The distribution of patients’ demographic and injury-related characteristics is presented in detail in Table 1.

With respect to in-hospital mortality during the initial admission, no statistically significant difference was observed between adult and geriatric patient populations (*p* = 0.764). Conversely, a significant divergence in one-year post-injury mortality was observed between the groups (*p* < 0.001). In the adult cohort, the one-year mortality rate was 4.7% (*n* = 12 patients), whereas in the geriatric cohort, the one-year mortality rate was calculated as 16.7% (*n* = 15 patients) (Table 2).

Baseline demographic, injury-related, clinical, and laboratory characteristics were compared between survivors and non-survivors separately in the adult and geriatric cohorts, according to both in-hospital and one-year mortality.

In the adult cohort, a higher ISS (*p* < 0.001) was significantly associated with in-hospital mortality following initial admission, whereas no significant associations were observed between mortality and other demographic characteristics or injury-related variables (*p* > 0.05). In contrast, within the geriatric cohort, male gender (*p* = 0.023), high-energy trauma (*p* = 0.011), and higher ISS (*p* < 0.001) were significantly associated with in-hospital mortality. ICU admission was significantly more prevalent among adult patients who died during the initial hospitalization than among survivors (*p* < 0.001). However, no significant difference was observed in the geriatric cohort (*p* = 0.576). Among patients admitted to the ICU, geriatric patients who did not survive had a significantly longer ICU stay (*p* = 0.006). With respect to laboratory parameters at initial presentation, leukocytosis (*p* = 0.018), elevated monocyte count (*p* = 0.001), and hypoalbuminemia (*p* < 0.001) were significantly associated with in-hospital mortality in the adult cohort. In the geriatric cohort, however, higher lymphocyte count (*p* < 0.001), lower NLR (*p* = 0.011), lower MLR (*p* = 0.005), lower PLR (*p* = 0.001), and elevated creatinine levels (*p* = 0.006) were found to be significantly associated with in-hospital mortality following initial admission (Table 3).

When parameters affecting one-year mortality after injury were evaluated, in the adult cohort, a significant association was identified between higher ISS (*p* < 0.001) and prolonged LOS during the initial hospitalization (*p* = 0.049) and one-year mortality rates. In the geriatric cohort, advanced age (*p* = 0.014), the need for major surgery at initial admission (*p* = 0.008), higher ISS (*p* = 0.002), and prolonged LOS in the hospital (*p* = 0.005) were found to be significantly associated with one-year mortality. In both the adult and geriatric cohorts, patients with a higher one-year mortality rate exhibited a greater probability of requiring ICU admission during the initial hospitalization (*p* < 0.001 and *p* = 0.010, respectively). Among patients admitted to the ICU, adult patients who died within one year exhibited a significantly longer ICU stay than survivors (*p* = 0.013). With respect to laboratory parameters, among the adults, lower hemoglobin (*p* = 0.017), lower hematocrit (*p* = 0.027), elevated monocyte count (*p* = 0.014), and hypoalbuminemia (*p* < 0.001) at initial admission were significantly associated with one-year mortality. Among the geriatric patients, lower hemoglobin (*p* = 0.015), lower hematocrit (*p* = 0.005), lower platelet count (*p* = 0.007), elevated lymphocyte count (*p* = 0.026), lower NLR (*p* = 0.002), lower PLR (*p* = 0.006), elevated urea (*p* < 0.001), elevated creatinine (*p* < 0.001), and hypoalbuminemia (*p* < 0.001) at the initial admission were significantly associated with one-year mortality (Table 4).

**Figure 1:**
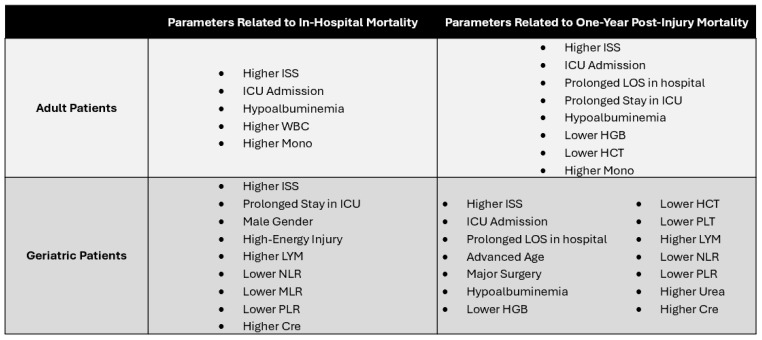
Summary of factors associated with in-hospital and one-year mortality in adult and geriatric patients.

When the parameters affecting mortality were evaluated collectively, a higher ISS was found to be associated with both in-hospital and one-year mortality in both adult and geriatric cohorts. Hypoalbuminemia and ICU admission were associated with in-hospital mortality in adults and with one-year mortality in both adult and geriatric patients. Prolonged hospital LOS was associated with one-year mortality in both groups. Higher LYM, lower NLR, lower PLR, and higher Cre levels were not found to be associated with post-traumatic mortality in adults; however, they were associated with both in-hospital and one-year mortality in geriatric patients. Higher Mono was found to be associated with both in-hospital and one-year mortality in adult patients. In addition, lower HGB and HCT levels were associated with one-year mortality in both adult and geriatric cohorts. (Details are provided in Table 3 and Table 4; a summary is presented in Figure 1).

The secondary subgroup analysis, which was restricted to patients with ISS > 15, yielded findings that were consistent with those of the primary analysis. The similar laboratory parameters and derived ratios remned significantly associated with mortality (see Table 5). The corresponding descriptive statistics for the overall study cohort are reported in Table 3 and Table 4.

## 4. Discussion

The primary objective of this multidisciplinary cohort study was to compare the clinical and laboratory factors associated with short- and long-term mortality after multisystem trauma in adult and geriatric patients, and to explore laboratory findings consistent with immunosenescence in geriatric patients after multisystem trauma. A key methodological strength of the study lies in the strict use of variables obtained at the time of initial emergency department presentation at the index hospital. Consequently, the findings provide a comprehensive overview of the early systemic response to trauma and facilitate a comparative analysis of age-related disparities in the prognostic significance of admission laboratory parameters. The most significant finding of the study was the marked age-dependent divergence in post-traumatic survival. While in-hospital mortality rates were comparable between adult and geriatric patients, one-year mortality rates were significantly higher in the geriatric cohort. Furthermore, higher ISS, prolonged hospital and ICU stay, various hematological indices and ratios, and hypoalbuminemia demonstrated differing prognostic significance between adult and geriatric patients. Collectively, the observed pattern of attenuated inflammatory laboratory findings despite increased long-term mortality in older adults may be consistent with the effects of immunosenescence and diminished immune reserve. Importantly, this pattern remained consistent in the secondary analysis restricted to patients with severe trauma (ISS > 15), suggesting that the observed age-related inflammatory profile was preserved even under conditions of greater physiological stress.

In the present study, in-hospital mortality rates among geriatric trauma patients were found to be comparable to those observed among the adult patients (*p* = 0.764). However, a significant increase in one-year mortality rates was observed (*p* < 0.001). This finding is consistent with previous studies showing that elderly trauma patients, although they generally survive the acute phase, experience excess mortality during the subsequent follow-up period [21]. This phenomenon of delayed mortality can be attributed to the diminished physiological reserves, whereby trauma initiates a progressive deterioration characterized by immobility-related complications, sepsis, and exacerbation of underlying chronic diseases [22,23]. A notable limitation of the present study is the absence of a comprehensive evaluation of baseline comorbidities and medication use. In older adults with multisystem trauma, long-term outcomes are influenced not only by the index injury but also by chronic diseases, frailty, and treatment-related factors such as polypharmacy [24]. Due to the retrospective nature of the study, which spanned a considerable duration, patients were managed by physicians from various specialties. Consequently, there was an inconsistency in the availability of standardized information regarding the comorbidity burden and medication history. As a result, the impact of pre-existing diseases could not be quantified using validated comorbidity indices. Furthermore, the absence of detailed medication data should be acknowledged. Drugs frequently prescribed to older adults, including corticosteroids, immunosuppressive agents, anticoagulants, and other long-term therapies, have the potential to substantially influence leukocyte counts, inflammatory cell ratios, and renal function parameters measured at admission. Additionally, mounting evidence suggests that the presence of multiple chronic conditions and the use of multiple medications (which may be termed “polypharmacy”) may interact with the process of immunosenescence and inflammaging. This interaction may influence immune responses that extend beyond the effects of chronological aging alone. Therefore, although the laboratory profile observed in our geriatric cohort is compatible with an immunosenescent phenotype, it should not be interpreted as evidence that age-related immune remodeling was the sole underlying mechanism. Instead, the observed alterations are likely the result of multiple interacting factors, including biological aging, chronic disease burden, frailty, and medication exposure. It is imperative that future prospective studies incorporating validated comorbidity indices, frailty assessments, and detailed medication histories be conducted to more accurately delineate the independent contribution of each variable.

In the present cohort, a comprehensive evaluation was conducted on the relationship between injury-related characteristics and mortality. The overall severity and extent of injury were assessed using the Injury Severity Score. A systematic analysis was conducted on injury-related variables, including injury energy, the necessity for major surgery, and the duration of hospital and intensive care unit stays. In accordance with extant literature [21,25,26] and clinical expectations, greater injury severity and burden, as indicated by higher ISS values, exhibited a significant association with both short-term (in-hospital) and long-term (one-year) mortality across both adult and geriatric populations. Furthermore, a correlation was identified between male gender, high-energy injury, and geriatric in-hospital mortality, similar to the literature [27]. When examining the mechanisms underlying the association between trauma characteristics and mortality, the observed relationships involving higher ISS, male gender, and high-energy injury emphasize the critical importance of the initial injury burden and the severity of early post-traumatic critical illness. The correlation between male gender and elevated short-term mortality rates may be partly attributable to sociocultural variations in activity patterns and trauma exposure among older adults within our population. For instance, elderly men may maintain higher levels of engagement in occupational or social activities, thereby increasing their exposure to more severe trauma mechanisms. However, this hypothesis warrants further investigation [28,29]. Prolonged hospital length of stay was associated with one-year mortality in both age groups. Conversely, ICU-related findings differed between the adult and geriatric cohorts. Because most patients did not require intensive care, reporting ICU length of stay for the entire cohort would have resulted in a median of zero days, providing little clinically meaningful information. Therefore, ICU admission and ICU length of stay among admitted patients were analyzed separately. ICU admission was not associated with in-hospital mortality in geriatric patients, whereas it was significantly associated with in-hospital mortality in adults and with one-year mortality in both age groups. In contrast, the relationship between ICU length of stay and mortality varied by age. Geriatric patients who died during their initial hospitalization had significantly longer ICU stays than survivors, whereas prolonged ICU stay was associated with one-year mortality only in the adult cohort. These findings may reflect age-related differences in physiological reserve, resilience to severe trauma, and recovery during critical illness. The prolonged ICU stay observed among geriatric non-survivors is also consistent with impaired physiological recovery associated with immunosenescence. However, given the limited number of mortality events, these findings should be interpreted with caution. The relationship between surgical treatment and survival appeared to differ according to age group. While major surgery was not significantly associated with mortality in adult patients, it demonstrated a significant association with one-year mortality in geriatric patients (*p* = 0.008). A plausible explanation for this phenomenon is that older adults may possess a diminished physiological reserve, which renders them less capable of tolerating the combined stresses of severe trauma, major surgery, and anesthesia. This diminished reserve predisposes them to postoperative deterioration, which can adversely affect long-term survival [30,31]. Furthermore, advanced age itself may directly contribute to mortality through intrinsic physiological vulnerability. Indeed, in the present study, increasing age was found to be significantly associated with one-year mortality among geriatric patients [21].

When laboratory parameters were examined as a whole, markers of metabolic and renal reserve offered valuable prognostic information. Among these, lower albumin levels at admission were significantly associated with in-hospital mortality in adults and with one-year mortality in both adult and geriatric patients. In the context of acute trauma, albumin functions not only as a nutritional marker but also as a negative acute-phase reactant. It has been observed to decrease in response to systemic inflammation and capillary leakage. Therefore, low albumin levels at the initial presentation may be indicative of both impaired baseline physiological reserve and a heightened early systemic response to injury. This heightened response is likely to contribute to the strong association between low albumin levels and subsequent mortality [32]. Moreover, elevated admission creatinine levels were associated with both in-hospital and one-year mortality in geriatric patients, whereas elevated urea levels were associated with one-year mortality only. A possible explanation is the limited renal reserve of the aging population, in which trauma-related hypoperfusion may more readily lead to clinically meaningful renal injury. In this setting, even transient acute kidney dysfunction may influence long-term outcome after the initial traumatic event [33]. Similarly, lower hemoglobin and hematocrit values at admission were associated with one-year mortality in both adult and geriatric patients, indicating that the severity of the early hemorrhagic and ischemic burden may have consequences that persist well beyond the acute phase of care [34].

One of the most noteworthy findings of this study was the age-dependent divergence in early systemic inflammatory patterns. In the adult population, elevated leukocyte counts were associated with in-hospital mortality, whereas elevated monocyte counts were associated with both in-hospital and one-year mortality. These findings suggest a more typical hyperinflammatory response to severe trauma. In younger patients, this early inflammatory activation may signify the classical systemic inflammatory response, in which excessive leukocyte recruitment and activation can contribute to secondary tissue damage and subsequent organ failure [35]. Conversely, geriatric patients exhibited an inflammatory profile that deviated considerably from the established paradigm observed in trauma populations. In geriatric patients, elevated lymphocyte counts, along with lower NLR and PLR, were associated with both short-term and long-term mortality, whereas lower MLR was associated only with in-hospital mortality. This observation appears contradictory to the broader trauma literature, where higher NLR is typically interpreted as a marker of greater physiological stress and poor prognosis [36,37]. A plausible explanation for these findings is that immunosenescence-associated immune remodeling limits the ability of older adults to mount the expected neutrophil-predominant inflammatory response after severe injury. As a result, patients at the highest risk of adverse outcomes may present with a relatively attenuated inflammatory profile rather than the hyperinflammatory response typically observed in younger adults [38]. This combination of blunted inflammatory activation and increased long-term mortality is consistent with age-related immune dysfunction and diminished immune reserve.

Age-related impairment of neutrophil function, including reduced chemotaxis and phagocytic capacity, may further contribute to this altered response and increase susceptibility to delayed infectious mortality [39]. These mechanisms are characteristic of immunosenescence, which affects both innate and adaptive immunity [40]. Our findings align with this concept, as geriatric patients exhibited a relatively attenuated inflammatory profile despite significantly higher one-year mortality. Similar observations have been reported in geriatric fracture populations, where atypical inflammatory marker patterns, particularly nonclassical NLR profiles, were associated with increased mortality risk [41]. These findings suggest that the prognostic interpretation of inflammatory markers should be age specific. In older adults with severe trauma, a lower or apparently normal NLR may not necessarily indicate physiological stability but instead reflect immunosenescence-related immune dysfunction. Notably, restricting the analysis to patients with severe injuries (ISS > 15) did not materially change the observed associations, indicating that the distinct inflammatory profile of the geriatric cohort persisted even under greater physiological stress. Although causality cannot be established, these results further support the hypothesis that age-related alterations in the post-traumatic immune response contribute to the unique inflammatory profile observed in older adults.

Previous studies in both general trauma populations and geriatric orthopedic trauma have consistently reported that elevated NLR is associated with worse outcomes and increased mortality [42,43,44,45]. In contrast, we observed that lower NLR and PLR, together with higher lymphocyte counts, were associated with increased mortality in the geriatric cohort. This discrepancy may reflect age-related alterations in immune function rather than a conventional hyperinflammatory response, supporting the hypothesis that immunosenescence and diminished immune reserve modify the inflammatory response to severe trauma in older adults.

The inflammatory profile observed in our geriatric cohort should be interpreted in the context of age-related factors. As previously mentioned, multimorbidity, frailty, and polypharmacy are highly prevalent in older adults and can independently influence inflammatory biomarkers, immune cell populations, and clinical outcomes following trauma. Therefore, while our findings are consistent with immunosenescence, they cannot be attributed solely to age-related immune remodeling. Rather, the observed laboratory alterations likely reflect the combined effects of biological aging and associated clinical factors. Future prospective studies incorporating validated frailty assessments, comorbidity indices, and detailed medication histories are needed to better delineate the independent contribution of immunosenescence to trauma outcomes.

It is imperative to acknowledge several limitations when interpreting the present findings. Firstly, the retrospective and single-center nature of the study may reduce generalizability. Secondly, a standardized comorbidity score, such as the Charlson Comorbidity Index [46], could not be comprehensively integrated into the analysis. This is particularly significant in the context of geriatric trauma, where the presence of chronic disease and frailty significantly impacts patient outcomes. The absence of standardized information regarding baseline comorbidities and medication histories is a significant limitation because polypharmacy can greatly impact the metabolism of elderly patients. As chronic diseases and medications such as corticosteroids, immunosuppressive agents, and anticoagulants may independently affect both one-year mortality and laboratory biomarkers measured at admission, the observed associations should be interpreted with caution. Thirdly, the analysis was intentionally constrained to laboratory parameters obtained at the initial emergency department presentation. While this design enabled our focus on baseline physiological and inflammatory status, it precluded assessment of dynamic biomarker trajectories over time, including changes in indices such as delta-NLR [47]. Fourthly, the study cohort included patients with a broad range of injury severity (minimum ISS = 4), which may have introduced clinical heterogeneity. However, a sensitivity analysis restricted to patients with ISS > 15 yielded findings consistent with the primary analysis, suggesting that the inclusion of patients with less severe injuries was unlikely to have materially influenced the overall conclusions. Nevertheless, injury severity heterogeneity remains an inherent limitation of the study.

In terms of methodological perspective, the inability to perform multivariable logistic regression analysis represents a significant methodological limitation. Although injury severity is an important determinant of post-traumatic mortality, multivariable regression analyses could not be performed because the number of mortality events became limited after stratification into adult and geriatric cohorts, thereby increasing the risk of model overfitting. Therefore, the observed relationships between laboratory parameters and mortality should be interpreted as associations rather than independent prognostic effects. Nevertheless, the sensitivity analysis restricted to patients with ISS > 15 yielded findings that were consistent with the primary analysis, suggesting that the inclusion of patients with less severe injuries was unlikely to have materially influenced the overall conclusions. Furthermore, the absence of detailed longitudinal survival data represents another limitation of this study. Because only the binary survival status at the one-year follow-up was available, rather than the exact time from injury to death, time-to-event analyses such as Kaplan–Meier survival estimation and Cox proportional hazards regression could not be performed. Future studies incorporating precise survival-time data may provide a more comprehensive understanding of the temporal relationship between inflammatory biomarkers and post-traumatic mortality. In addition, standardized effect sizes and odds ratios were not reported because the analyses were based primarily on non-parametric comparisons of continuous variables. Finally, a substantial number of statistical comparisons were conducted without formal correction for multiple testing. While this approach was deemed suitable for the exploratory nature of the study, it does increase the probability of type I error. Consequently, the observed associations should be interpreted as hypothesis-generating and require confirmation in larger prospective studies.

## 5. Conclusions

Geriatric patients suffering from multisystem trauma have been shown to exhibit a significantly elevated one-year mortality rate, despite maintaining a comparable in-hospital mortality rate to that of younger adults. The admission laboratory parameters reflecting renal function, nutritional status, hematological indices, and the systemic inflammatory response exhibited age-related differences in their associations with mortality. Notably, older patients exhibited a comparatively diminished inflammatory response despite demonstrating poorer long-term survival outcomes. In contrast, younger adults exhibited a more conventional hyperinflammatory response following trauma.

In the context of geriatric patients, lower NLR and PLR values were found to be associated with both short- and long-term mortality, suggesting that non-elevated inflammatory indices may not necessarily indicate physiological stability in this demographic. Collectively, these findings may be consistent with age-related alterations in immune function and raise the possibility that immunosenescence contributes to the distinct relationship between inflammatory markers and outcomes in older trauma patients. Further prospective studies incorporating comprehensive comorbidity assessment, medication data, and validated measures of immune function are required to clarify these associations and determine their potential clinical relevance.

## Figures and Tables

**Table 1:** Demographic and injury-related characteristics of multisystem trauma patients.

		Number of Patients	Percentage (%)
Age * (years)		49 (18–95)	
Patient Group	Adult	255	73.9%
	Geriatric	90	26.1%
Gender	Female	116	33.6%
	Male	229	66.4%
Injury Energy	Low-Energy	90	26.1%
	High-Energy	255	73.9%
Injury Severity Score *		13 (4–57)	
LOS in Hospital at Initial Admission * (days)		5 (0–53)	
ICU Admission	No	259	75.1%
	Yes	86	24.9%
LOS in ICU Among Admitted * (days)		6 (1–43)	
Necessity of Major Surgery at Initial Admission	No	204	59.1%
	Yes	141	40.9%
In-hospital Survival during Initial Admission	No	14	4.1%
	Yes	331	95.9%
One-year Post-injury Survival	No	27	7.8%
	Yes	318	92.2%

*: Variables were presented as median and minimum–maximum range.

**Table 2:** Comparison of mortality rates by age group.

		In-Hospital Survival		*p*
		No	Yes	
Adult Patients	Count (N)	10	245	0.764
	% within group	3.9%	96.1%	
Geriatric Patients	Count (N)	4	86	
	% within group	4.4%	95.6%	
		One-year Post-injury Survival		
Adult Patients	Count (N)	12	243	<0.001
	% within group	4.7%	95.3%	
Geriatric Patients	Count (N)	15	75	
	% within group	16.7%	83.3%	

*p*: Statistical significance value.

**Table 3:** Comparison of clinical and laboratory parameters between survivors and non-survivors according to in-hospital mortality.

				In-Hospital Survival After Initial Admission		*p*
				No (Mortality)	Yes (Survival)	
Age (years)			Adult	32 (30–51)	39 (18–64)	0.428
			Geriatric	70.5 (65–76)	77 (65–95)	0.104
Gender *	Adult		Female	2 (3.2%)	60 (96.8%)	0.746
			Male	8 (4.1%)	185 (95.9%)	
	Geriatric		Female	0	54 (100%)	0.023
			Male	4 (11.1%)	32 (88.9%)	
Injury Energy *	Adult		Low-Energy	0	30 (100%)	0.612
			High-Energy	10 (4.4%)	215 (95.6%)	
	Geriatric		Low-Energy	0	60 (100%)	0.011
			High-Energy	4 (13.3%)	26 (86.7%)	
Major Surgery *	Adult		No	8 (5.3%)	142 (94.7%)	0.204
			Yes	2 (1.9%)	103 (98.1%)	
	Geriatric		No	2 (3.7%)	52 (96.3%)	0.676
			Yes	2 (5.6%)	34 (94.4%)	
Injury Severity Score			Adult	38 (33–57)	14 (4–45)	<0.001
			Geriatric	51 (45–57)	13 (4–41)	<0.001
LOS in Hospital (days)			Adult	7 (1–20)	5 (0–53)	0.265
			Geriatric	22 (1–45)	4 (0–45)	0.327
ICU Admission		Adult	No	2 (1%)	193 (99%)	<0.001
			Yes	8 (13.3%)	52 (86.7%)	
		Geriatric	No	2 (3.1%)	62 (96.9%)	0.576
			Yes	2 (7.7%)	24 (92.3%)	
LOS in ICU Among Admitted Patients * (days)			Adult	8 (2–20)	6 (1–25)	0.145
			Geriatric	43 (43–43)	4 (1–40)	0.006
Laboratory Parameters						
Hemoglobin/HGB (g/dL)			Adult	14 (3.0–15.2)	14.2 (7.1–18.8)	0.131
			Geriatric	13 (12.2–13.8)	12.3 (3.7–15.6)	0.336
Hematocrit/HCT (%)			Adult	40.5 (10.1–45.9)	42 (2.5–53.1)	0.179
			Geriatric	39.5 (36.8–42.2)	37.95 (24.5–47.3)	0.367
Platelet/PLT (×10^9^/L)			Adult	286 (113–287)	266 (86–589)	0.769
			Geriatric	249 (239–259)	253 (122–599)	0.828
Leukocyte/WBC (×10^9^/L)			Adult	23.83 (2.38–28.45)	13.4 (2.79–30.33)	0.018
			Geriatric	12.5 (12.37–12.62)	11.51 (2.02–40.75)	0.671
Neutrophil/Neu (×10^9^/L)			Adult	19.33 (1.66–25.28)	10 (1.54–25.64)	0.088
			Geriatric	8.37 (7.53–9.21)	9.46 (1.3–38.38)	0.741
Lymphocyte/LYM (×10^9^/L)			Adult	2.93 (0.07–6.5)	1.92 (0.29–7.55)	0.202
			Geriatric	3.36 (2.5–4.22)	1.16 (0.31–6.13)	<0.001
Monocyte/Mono (×10^9^/L)			Adult	1.12 (0.36–1.65)	0.58 (0.14–1.65)	0.001
			Geriatric	0.43 (0.25–0.6)	0.54 (0–1.74)	0.289
Neutrophil-to-Lymphocyte Ratio/NLR			Adult	6.61 (1.29–23.71)	4.58 (0.78–40.45)	0.519
			Geriatric	2.64 (1.83–3.6)	7.01 (0.62–67.03)	0.011
Monocyte-to-Lymphocyte Ratio/MLR			Adult	0.41 (0.13–5.14)	0.29 (0.05–1.76)	0.221
			Geriatric	0.14 (0.06–0.22)	0.44 (0–1.97)	0.005
Platelet-to-Lymphocyte Ratio/PLR			Adult	97.72 (44–1612.86)	134.38 (39.48–941.03)	0.200
			Geriatric	76.48 (61.4–95.56)	230.55 (47.32–923.24)	0.001
Urea (mg/dL)			Adult	37 (26.9–51.1)	35.1 (14.9–75.1)	0.941
			Geriatric	52.5 (48.9–56.1)	48.9 (18.9–186.1)	0.502
Creatinine (mg/dL)			Adult	0.81 (0.5–1.57)	0.86 (0.33–8.8)	0.841
			Geriatric	1.6 (1.31–1.89)	0.85 (0.4–3.73)	0.006
Albumin (g/L)			Adult	36 (35–38)	43 (25–52)	<0.001
			Geriatric	36 (33–39)	40 (25–52)	0.079

*p*: Statistical significance value. Continuous variables are presented as medians (range: minimum–maximum). Variables marked with an asterisk (*) are presented as frequency (percentage).

**Table 4:** Comparison of clinical and laboratory parameters between survivors and non-survivors according to one-year mortality.

				One-Year Post-Injury Survival		*p*
				No (Mortality)	Yes (Survival)	
Age (years)			Adult	34.5 (30–51)	39 (18–64)	0.427
			Geriatric	85 (65–95)	75 (65–92)	0.014
Gender *	Adult		Female	4 (6.5%)	58 (93.5%)	0.456
			Male	8 (4.1%)	185 (95.9%)	
	Geriatric		Female	6 (11.1%)	48 (88.9%)	0.083
			Male	9 (25%)	27 (75%)	
Injury Energy *	Adult		Low-Energy	0	30 (100%)	0.370
			High-Energy	12 (5.3%)	213 (94.7%)	
	Geriatric		Low-Energy	11 (18.3%)	49 (81.7%)	0.765
			High-Energy	4 (13.3%)	26 (86.7%)	
Major Surgery *	Adult		No	10 (6.7%)	140 (93.3%)	0.130
			Yes	2 (1.9%)	103 (98.1%)	
	Geriatric		No	4 (7.4%)	50 (92.6%)	0.008
			Yes	11 (30.6%)	25 (69.4%)	
Injury Severity Score			Adult	38 (33–57)	14 (4–45)	<0.001
			Geriatric	20 (8–57)	13 (4–34)	0.002
LOS in Hospital (days)			Adult	8 (1–29)	5 (0–53)	0.049
			Geriatric	10 (0–45)	3 (0–19)	0.005
ICU Admission		Adult	No	2 (1%)	193 (99%)	<0.001
			Yes	10 (16.7%)	50 (83.3%)	
		Geriatric	No	6 (9.4%)	58 (90.6%)	0.010
			Yes	9 (34.6%)	17 (65.4%)	
LOS in ICU Among Admitted Patients * (days)			Adult	9.5 (2–25)	5.5 (1–19)	0.013
			Geriatric	18 (1–43)	3 (1–16)	0.107
Laboratory Parameters						
Hemoglobin/HGB (g/dL)			Adult	11.85 (3–15.2)	14.2 (8.1–18.8)	0.017
			Geriatric	11.1 (7.1–13.8)	12.5 (3.7–15.6)	0.015
Hematocrit/HCT (%)			Adult	35.7 (10.1–45.9)	42.1 (2.5–53.1)	0.027
			Geriatric	33 (24.5–42.2)	38.4 (25.9–47.3)	0.005
Platelet/PLT (×10^9^/L)			Adult	286 (113–491)	266 (86–589)	0.475
			Geriatric	213 (169–295)	254 (122–599)	0.007
Leukocyte/WBC (×10^9^/L)			Adult	20.09 (2.38–28.45)	13.41 (2.79–30.33)	0.141
			Geriatric	11.44 (2.02–22.59)	12.2 (5.32–40.75)	0.395
Neutrophil/Neu (×10^9^/L)			Adult	13.81 (1.66–25.28)	10.04 (1.54–25.64)	0.312
			Geriatric	7.54 (1.3–18.7)	9.82 (2.66–38.38)	0.147
Lymphocyte/LYM (×10^9^/L)			Adult	2.25 (0.07–6.5)	1.93 (0.29–7.55)	0.536
			Geriatric	1.9 (0.49–4.22)	1.08 (0.31–6.13)	0.026
Monocyte/Mono (×10^9^/L)			Adult	1.07 (0.28–1.65)	0.59 (0.14–1.65)	0.014
			Geriatric	0.5 (0.03–1.74)	0.53 (0–1.36)	0.914
Neutrophil-to-Lymphocyte Ratio/NLR			Adult	6.11 (1.29–23.71)	4.56 (0.78–40.45)	0.468
			Geriatric	3.41 (1.83–10.19)	7.84 (0.62–67.03)	0.002
Monocyte-to-Lymphocyte Ratio/MLR			Adult	0.39 (0.13–5.14)	0.29 (0.05–1.76)	0.235
			Geriatric	0.25 (0.06–0.95)	0.44 (0–1.97)	0.095
Platelet-to-Lymphocyte Ratio/PLR			Adult	125.09 (44.02–1612.86)	133.67 (39.48–941.03)	0.838
			Geriatric	96.92 (61.4–434.49)	239.41 (47.32–923.24)	0.006
Urea (mg/dL)			Adult	37.5 (26.9–51.1)	34.9 (14.9–75.1)	0.446
			Geriatric	67.9 (48.9–186.1)	46.9 (18.9–103.1)	<0.001
Creatinine (mg/dL)			Adult	0.97 (0.5–3.4)	0.86 (0.33–8.8)	0.420
			Geriatric	1.44 (0.58–3.73)	0.83 (0.4–2.48)	<0.001
Albumin (g/L)			Adult	35.5 (25–38)	43 (29–52)	<0.001
			Geriatric	37 (26–40)	41 (25–52)	<0.001

*p*: Statistical significance value. Continuous variables are presented as medians (range: minimum–maximum). Variables marked with an asterisk (*) are presented as frequency (percentage).

**Table 5:** Clinical and laboratory parameters associated with in-hospital and one-year mortality in patients with an Injury Severity Score > 15.

		In-Hospital Mortality	One-Year Post-Injury Mortality
Hemoglobin/HGB (g/dL)	Adult	0.385	0.072
	Geriatric	0.393	0.271
Hematocrit/HCT (%)	Adult	0.419	0.082
	Geriatric	0.248	0.374
Platelet/PLT (×10^9^/L)	Adult	0.770	0.447
	Geriatric	0.450	0.189
Leukocyte/WBC (×10^9^/L)	Adult	0.048	0.334
	Geriatric	0.790	0.413
Neutrophil/Neu (×10^9^/L)	Adult	0.177	0.635
	Geriatric	0.340	0.145
Lymphocyte/LYM (×10^9^/L)	Adult	0.275	0.582
	Geriatric	<0.001	0.010
Monocyte/Mono (×10^9^/L)	Adult	0.003	0.040
	Geriatric	0.208	0.497
Neutrophil-to-Lymphocyte Ratio/NLR	Adult	0.770	0.788
	Geriatric	0.002	<0.001
Monocyte-to-Lymphocyte Ratio/MLR	Adult	0.286	0.303
	Geriatric	0.001	0.005
Platelet -to-Lymphocyte Ratio/PLR	Adult	0.246	0.912
	Geriatric	0.002	0.005
Urea (mg/dL)	Adult	0.786	0.320
	Geriatric	1.000	0.058
Creatinine (mg/dL)	Adult	0.836	0.410
	Geriatric	0.038	0.008
Albumin (g/L)	Adult	<0.001	<0.001
	Geriatric	0.142	0.008

Only *p* values are presented in this table. Descriptive statistics for the overall study cohort are provided in Table 3 and Table 4.

## Data Availability

The datasets generated and/or analyzed during the current study are stored in a private repository and are not publicly available; however, they may be obtained from the corresponding author upon reasonable request.

## Abbreviations

The following abbreviations are used in this manuscript:

STROBE	Strengthening the Reporting of Observational Studies in Epidemiology
AIS	Abbreviated Injury Scale
ISS	Injury Severity Score
ICU	Intensive Care Unit
LOS	Length of Stay
iHM	In-Hospital Mortality
OYM	One-Year Mortality
HGB	Hemoglobin
HCT	Hematocrit
PLT	Platelet Count
WBC	Leukocyte/White Blood Cell Count
Neu	Neutrophil Count
LYM	Lymphocyte Count
Mono	Monocyte Count
Cr	Serum Creatinine
Alb	Serum Albumin
NLR	Neutrophil-To-Lymphocyte Ratio
MLR	Monocyte-To-Lymphocyte Ratio
PLR	Platelet-To-Lymphocyte Ratio
